# How Does Silicon Mediate Plant Water Uptake and Loss Under Water Deficiency?

**DOI:** 10.3389/fpls.2018.00281

**Published:** 2018-03-05

**Authors:** Daoqian Chen, Shiwen Wang, Lina Yin, Xiping Deng

**Affiliations:** ^1^State Key Laboratory of Soil Erosion and Dryland Farming on the Loess Plateau, Institute of Soil and Water Conservation, Northwest A&F University, Yangling, China; ^2^College of Crop Science, Fujian Agriculture and Forestry University, Fuzhou, China; ^3^Institute of Soil and Water Conservation, Chinese Academy of Sciences and Ministry of Water Resources, Yangling, China

**Keywords:** silicon, water status, water balance, drought, salt stress, transpiration, water uptake

## Abstract

In plants, water deficiency can result from a deficit of water from the soil, an obstacle to the uptake of water or the excess water loss; in these cases, the similar consequence is the limitation of plant growth and crop yield. Silicon (Si) has been widely reported to alleviate the plant water status and water balance under variant stress conditions in both monocot and dicot plants, especially under drought and salt stresses. However, the underlying mechanism is unclear. In addition to the regulation of leaf transpiration, recently, Si application was found to be involved in the adjustment of root hydraulic conductance by up-regulating aquaporin gene expression and concentrating K in the xylem sap. Therefore, this review discusses the potential effects of Si on both leaf transpiration and root water absorption, especially focusing on how Si modulates the root hydraulic conductance. A growing number of studies support the conclusion that Si application improves plant water status by increasing root water uptake, rather than by decreasing their water loss under conditions of water deficiency. The enhancement of plant water uptake by Si is achievable through the activation of osmotic adjustment, improving aquaporin activity and increasing the root/shoot ratio. The underlying mechanisms of the Si on improving plant water uptake under water deficiency conditions are discussed.

## Introduction

Silicon (Si) is the second most abundant element in soil. Plants generally take up Si in the form of soluble monosilicic acid H_4_SiO_4_, which normally ranges from 0.1 to 0.6 mM in the soil solution (Ma and Yamaji, 2006). All terrestrial plants contain Si in their tissues although the contents of Si varies considerably among species, ranging from 0.1 to 10% Si on a dry weight basis (Ma and Yamaji, 2006; Cornelis et al., 2010; Sahebi et al., 2015). Si has not been recognized as an essential element for plant growth, it does exert beneficial effects for many plant species, including both monocots and dicots (Ma and Yamaji, 2015). Indeed, Si seems to alleviate the detrimental effects of various stresses, including drought, salinity, heat, cold, metal toxicity, nutrient imbalance, plant pathogens, and insect pests (Liang et al., 2007; Guntzer et al., 2012; Hernandez-Apaolaza, 2014; Zhang et al., 2014; Meharg and Meharg, 2015; Vivancos et al., 2015; Guo et al., 2016; Reynolds et al., 2016).

Water deficiency is one of the major environmental constraints of plant growth and crop productivity (Chaves and Oliveira, 2004; Verslues et al., 2006). Plant water deficiency may result from a shortage of water in soil (drought) or from an obstacle to water uptake (physiological drought). Plant water deficiency can also be caused by the excessive high vapor pressure deficit in the atmosphere, which results in higher rates of water loss via transpiration than the rates of water transport to the leaves (Mahajan and Tuteja, 2005). In these cases, plant water status is disturbed, resulting in disruption of important metabolic processes and reduction in growth rates (Verslues et al., 2006). Hence, investigating mechanisms of plants’ ability to tolerate water stress may lead to an understanding of how to increase water stress resistance. Recently, improvement of plant resistance to drought, osmotic, and salt stresses have been widely observed after the addition of Si to the growth medium (Zhu and Gong, 2014; Rizwan et al., 2015; Coskun et al., 2016; Helaly et al., 2017).

Several different aspects are involved in Si-improved plants’ resistance to drought or salt stress, including maintenance of nutrient balance, promotion of photosynthetic rate, increasing antioxidant capacity, and sequestration of toxic ions (Ma, 2004; Liang et al., 2007; Sacała, 2009; Zhu and Gong, 2014; Rizwan et al., 2015). Moreover, various compounds of Si, including 1–2 mM Na_2_SiO_3_, K_2_SiO_3_ or H_2_SiO_3_, either applied in the soil or the nutrient solution, are showed to improve the water status of plants experiencing drought or salt stress (Romero-Aranda et al., 2006; Sacała, 2009; Liu et al., 2014, 2015). In addition, it has been reported that supplement with 1 mM H_2_SiO_3_ in the nutrient solution can alleviate K deficiency, which also causes tissue dehydration (Chen et al., 2016). A variety of beneficial effects of Si application could be ascribed to the alleviation of problematic water status in those studies by decreasing the transpiration rate, increasing the osmotic adjustment capacity, or increasing water uptake (Liang et al., 2007; Sacała, 2009; Zhu and Gong, 2014; Rizwan et al., 2015). In this review, we address recent results that are relevant to the Si effect, and assess what they mean for the interpretation of how Si improves plant water status and enables the maintenance of plant water balance under water deficiency condition.

## Silicon Contributes to Alleviation of Plant Water Status Under Stress Conditions

A common consequence of several abiotic stresses is the disturbance of plant water status. Abiotic stresses, such as drought, salinity, and freezing have a common impact on plant cells in decreasing the availability of water (Mahajan and Tuteja, 2005; Verslues et al., 2006), quantified as a decrease in plant water potential and relative water content. Conversely, maintenance of higher relative water contents indicates a better water status (Verslues et al., 2006).

Under drought stress, the beneficial effect of Si on plant water status has been extensively examined in various plant species, including sorghum (Hattori et al., 2007; Yin et al., 2013; Ahmed et al., 2014), wheat (Gong and Chen, 2012), maize (Amin et al., 2014), rice (Ming et al., 2012), cucumber (Ma et al., 2004), Kentucky Bluegrass (Saud et al., 2014), canola (Habibi, 2014), sunflower (Gunes et al., 2008), chickpea (Gunes et al., 2007), soybean (Shen et al., 2010), alfalfa (Liu and Guo, 2013), and tomato (Shi et al., 2016). The improvements of relative water content and/or water potential by Si application occurred under both polyethylene glycol-induced osmotic stress (Hattori et al., 2007; Ming et al., 2012) and potted soil drought conditions (Gong et al., 2003; Amin et al., 2014). In addition, it has been showed that in the leaves of Si-treated wheat, both relative water contents and the water potential were maintained to a greater extent compared to that without Si-treatment, suggesting that Si could also be used to improve the water status of wheat under field drought conditions (Gong and Chen, 2012).

Under salt stress condition, the beneficial role of Si in mitigating the adverse effects of salinity by preventing root Na^+^ uptake and/or its transport from roots to shoots has been widely reported (Liang et al., 2007; Savvas et al., 2007, 2009; Zhu and Gong, 2014; Savvas and Ntatsi, 2015). In addition to ion toxicity, high concentrations of salts in solution also cause osmotic stress in plants, because they limit the availability of water, affecting water status and leaf growth (Munns and Tester, 2008). Chen et al. (2014) reported that Si could alleviate the salt stress in both two phases of growth inhibition, with the alleviative effects being more pronounced in the osmotic stress phase than ion toxicity phase. Moreover, Si application is widely reported to improve the leaf relative water contents and/or leaf water potential under salt stress in wheat (Tuna et al., 2008), rice (Yeo et al., 1999), sorghum (Liu et al., 2015), maize (Parveen and Ashraf, 2010), tomato (Li et al., 2015), *Phaseolus vulgaris* (Zuccarini, 2008), sunflower (Ashraf et al., 2015), and cucumber (Wang et al., 2015). The only exception to these findings was the observation that Si decreased tomato leaf water potential under salt stress (Romero-Aranda et al., 2006). However, in this study, plant water content in salinized plants supplied with Si was 40% higher than in salinized plants without Si, and leaf turgor potential and net photosynthetic rates were much higher in salinized plants with Si. Therefore, in spite of the leaf water potential, it can be concluded that Si improves the water status of tomato under salt stress.

A recent study in sorghum showed that Si could alleviate potassium (K) deficiency by improving plant water status (Chen et al., 2016). K is the most abundant cation in plants and plays a key role in osmotic processes that contribute to cellular turgor, photosynthesis, and transpiration (Wang and Wu, 2013). K is involved in regulating the plant water status, and severe K deficiency causes tissue dehydration (Kanai et al., 2011). Moreover, it has also been reported that Si could enhance freezing stress resistance in freezing-susceptible wheat cultivar by alleviating water-deficit stress that caused by freezing-induced cellular dehydration (Liang et al., 2008).

## Silicon Contributes to Maintaining Higher Transpiration Under Stress Conditions

Under normal growth conditions, water is absorbed by the roots and lost from the leaves, and plants keep a proper water balance by continuously adjusting these two processes (Maurel and Chrispeels, 2001). Under water deficient conditions, the plant’s first response is to avoid low water potential by adjust their water balance between root water uptake and leaf water loss (Luu and Maurel, 2005; Verslues et al., 2006). Plants can reduce leaf water loss by controlling the transpiration rate and also by decreasing their leaf area. Under normal growth conditions, only a few reports have shown that Si affects the transpiration rate. The pioneering researchers in this field reported that Si application can reduce the excessive leaf transpiration in rice and sugarcane under normal growth conditions; they postulated that this effect could be due to the reduction in transpiration rate through cuticular layers thickened by silica deposits (Yoshida, 1965; Savant et al., 1996). However, other researchers reported that rather than due to the thickness of cuticular layers, the reduced transpiration levels of Si-fed maize and rice were primarily due to the lower transpiration through stomatal pores (Agarie et al., 1998; Gao et al., 2004, 2006), which mainly ascribed to the turgor loss of guard cells originating from Si deposition and changing of the physical and mechanical properties of their cell walls (Ueno and Agarie, 2005; Savvas and Ntatsi, 2015). The reduced transpiration rates caused by Si were also reported in upland rice and cucumber in the absence of stress (Ma et al., 2004; Ming et al., 2012). Despite these reports, Si application has been found to have no effect on transpiration rates under normal growth conditions in the vast majority of studies (Hattori et al., 2005, 2009; Chen et al., 2011; Gong and Chen, 2012). We suspect that the conflicting results are due to species and genotypic variations since we have noticed that the effects of Si on reducing the transpiration rate under normal growth conditions tend to appear in the species and genotypes with high Si accumulation and high contribution of cuticular transpiration to total transpiration.

When plants first begin to experience drought stress, they decrease the leaf water loss mainly by decreasing the leaf transpiration rate through stomatal closure. Conflicting reports exist in the literature regarding the impact of Si on leaf transpiration rate. Maize leaf transpiration is reported to be decreased by Si in the studies of Gao et al. (2004, 2006) and Amin et al. (2014). Liu and Guo (2013) reported that Si application reduced both the transpiration rate and stomatal conductance but had no effect on photosynthetic rate in alfalfa under drought stress. Although, it has been reported that Si reduced the excessive leaf transpiration in rice under normal growth conditions (Savant et al., 1996; Agarie et al., 1998; Ming et al., 2012), the results of Chen et al. (2011) and Ming et al. (2012) showed that rice leaf transpiration was enhanced by Si when the plants were experiencing drought. Many other results on drought stressed plants have been shown to be consistent with enhanced leaf transpiration by Si application (Hattori et al., 2005; Sonobe et al., 2009; Chen et al., 2011; Gong and Chen, 2012; Pereira et al., 2013; Zhang et al., 2013; Liu et al., 2014; Saud et al., 2014; Kang et al., 2016). Under salt stress, the leaf transpiration rate has also been widely reported to be enhanced by Si (Yeo et al., 1999; Parveen and Ashraf, 2010; Liu et al., 2015; Wang et al., 2015; Mahmood et al., 2016; Qin et al., 2016). Also relevant are the findings of Chen et al. (2016), who reported that Si application enhances the transpiration of sorghum experiencing K-deficiency. Therefore, we conclude that Si application generally enhances transpiration of plants under various conditions of water stress.

## Silicon Enhances Root Water Uptake Under Stress Conditions

During water deficiency, the regulation of root water uptake, in some cases, may be more crucial to overcome stress injury than the regulation of leaf water loss (Aroca et al., 2012). Compared with the effect of Si on the leaf transpiration, fewer studies have focused on the impact of Si on root water uptake. Root water uptake capacity is represented by root hydraulic conductance (Steudle, 2000). Recently, improving root hydraulic conductance by Si application has been directly demonstrated in sorghum (Hattori et al., 2007; Sonobe et al., 2009, 2010; Liu et al., 2014), rye (Hattori et al., 2009), tomato (Shi et al., 2016), and cucumber (Wang et al., 2015; Zhu et al., 2015) under drought stress, salt stress and K deficiency conditions.

The extent of root hydraulic conductance depends on the driving force, root surface area, root anatomy, and root’s permeability to water (Steudle, 2000; Vandeleur et al., 2009; Sutka et al., 2011). A promotion of osmotic driving force by Si application has been observed in various studies. Sonobe et al. (2010) suggested that Si application leads to a strong water potential gradient through accumulation of soluble sugars and amino acids in the plant. A similar consequence of Si application was observed in rice (Ming et al., 2012) and canola (Habibi, 2014) under drought stress. Liu et al. (2015) reported that Si had no effect on osmotic potential of root xylem sap under osmotic stress although it increased root hydraulic conductance in sorghum (Liu et al., 2015). In the study of tomato under osmotic stress, water stress also did not cause the change in root osmotic potential in Si-treated plants (Shi et al., 2016). Under salt stress, Zhu et al. (2015) found that Si decreased root xylem osmotic potential via accumulation of soluble sugars in cucumber. Under K deficiency condition, Si was also seen to decrease the root xylem osmotic potential through accumulation of K in sorghum (Chen et al., 2016). Therefore, under those conditions, regulation of the osmotic driving force could play a central role in Si-mediated enhancement of water uptake.

In addition to driving force, aquaporins were reported to play a central role in regulating root water permeability in response to short term water stress (Maurel et al., 2008). Liu et al. (2014) firstly reported that Si-pretreatment significantly increased the expression of aquaporin genes, which in turn increased the root water uptake in sorghum under drought stress. Recently, Liu et al. (2015) and Zhu et al. (2015) also observed that Si application increased aquaporin expression in sorghum and cucumber under salt stress. In addition, Si can also increase aquaporin expression in sorghum under K deficiency (Chen et al., 2016). However, the expression of aquaporin genes was not significantly regulated (less than twofold) by Si application in tomato under water stress (Shi et al., 2016). It is worth noting here that only three aquaporin genes (*SlPIP1; 3*, *SlPIP1; 5*, and *SlPIP2; 6*) were studied in this tomato study. Furthermore, modulation of aquaporin transport activity can also occur at post-transcriptional level. It is speculated that increased root hydraulic conductance by Si under stress conditions may be partly ascribed to Si-induced reductions in oxidative stress and membrane damage (Li et al., 2015; Shi et al., 2016). Similarly, Liu et al. (2015) suggested that Si could enhance aquaporin activity by reducing H_2_O_2_ accumulation. Certainly, it can be concluded that regulation of aquaporin transport activity is involved in Si-induced enhancement of root hydraulic conductance under stress conditions. But whether it is a general mechanism for the enhancement of root hydraulic conductance under stress conditions requires further study.

When long-term water stress occurred, changes in root surface and anatomy may also be important for enhancing plant water uptake (Javot and Maurel, 2002). Under drought stress, Si-pretreatment has been reported to increase the root/shoot ratio, contributing to a higher ability of water uptake in sorghum (Hattori et al., 2005, 2009). The increased root/shoot ratio was also observed in other studies of sorghum (Ahmed et al., 2011a,b) and rice (Ming et al., 2012) under drought stress as well as cucumber under salt stress (Wang et al., 2015). These results suggest that Si-mediated modifications of root growth may also account for the increase in the water uptake ability in Si-treated plants. However, Liu et al. (2014) did not observe any Si-mediated changes in vessel diameter or vessel number of sorghum root under drought stress. And several researchers observed no effect of Si on the root/shoot ratio in other plant species under stress conditions (Gong et al., 2003; Gao et al., 2004; Sonobe et al., 2009; Chen et al., 2011; Shi et al., 2016). In summary, Si-mediated modification of root growth may enhance root water uptake under stress conditions, but this adjustment is not a common phenomenon to all plants and it remains unclear whether Si is directly involved in the modification of root growth or not. Further studies are needed to clarify how Si regulates root development under water deficient condition.

## Conclusion and Perspectives

**FIGURE 1:**
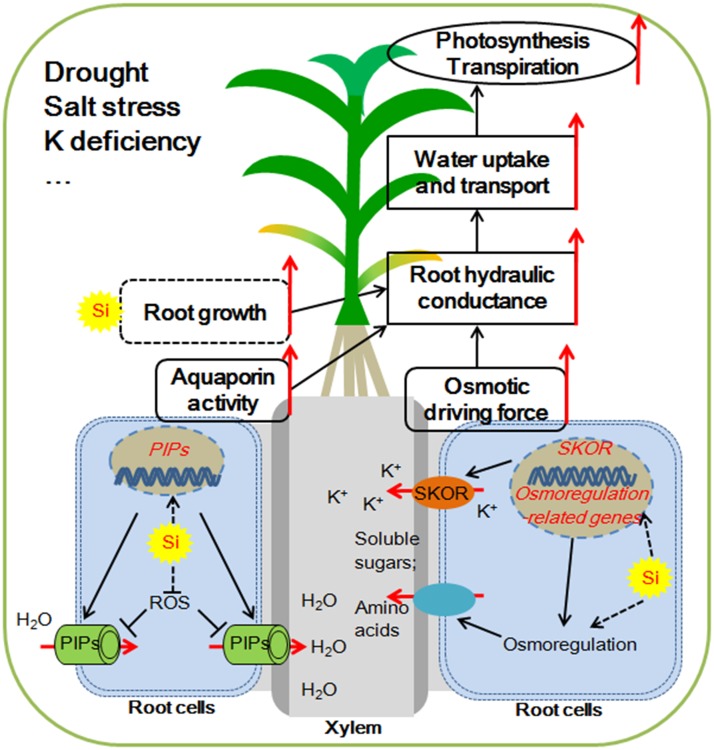
Possible mechanisms for silicon (Si) mediated water balance of plants experiencing water deficiency. (1) Si enhances the aquaporin activity by up-regulating the expression of *Plasma membrane Intrinsic Protein* (*PIP*) aquaporin genes and alleviating the ROS (reactive oxygen species)-induced aquaporin activity inhibition. (2) Si enhances the accumulation of soluble sugars and/or amino acids in the xylem sap by osmorugulation; Si activates the K^+^ translocation to xylem sap by the activation the expression of *SKOR* (*Stelar K^+^ Outward Rectifer*) gene. The osmolyte accumulations in the xylem sap increase the osmotic driving force. (3) Si might adjust the root growth and increase root/shoot ratio, which together with enhancement of aquaporin activity and osmotic driving force contribute to the improvement of root hydraulic conductance. The higher root hydraulic conductance results in increased uptake and transport of water, which helps to maintain a higher photosynthetic rate and improve plant resistance to water deficiency.

Water deficiency is one of the major environmental factors limiting the growth of plants and the production of crops. The investigations described here showed that Si application moderates the plant hydraulic properties by increasing the root water uptake, but not by decreasing their water loss under water deficient condition. As illustrated in **Figure 1**, the potential key mechanisms involved in Si-mediated enhancement of plant root water uptake under water deficiency include: (1) enhancement of the osmotic driving force via active osmotic adjustment; (2) improvement of aquaporin transport activity at both transcriptional and post-transcriptional levels; (3) modification of root growth and increasing root/shoot ratio (**Figure 1**).

Predictions of future global environmental changes point to an increase in both the severity and frequency of water stress in the near future. Therefore, genetic and biochemical manipulation of crops to increase their capacities of Si absorption, translocation and distribution from applied Si fertilizer should be considered as a preferable choice to improve crop production under water deficient condition. However, the mechanisms behind the beneficial effects of Si are still largely unknown. Hence, the mechanisms by which Si moderates the plant water status still need further investigation, especially regarding the molecular and biochemical basis by which Si regulates plant water uptake. In addition, the application of Si and its performance under field conditions still needs extensive investigation.

## Author Contributions

SW and DC wrote the manuscript. LY and XD helped in drafting the manuscript.

## Conflict of Interest Statement

The authors declare that the research was conducted in the absence of any commercial or financial relationships that could be construed as a potential conflict of interest.
